# Oral tongue leukoplakia: analysis of clinicopathological characteristics, treatment outcomes, and factors related to recurrence and malignant transformation

**DOI:** 10.1007/s00784-020-03735-1

**Published:** 2021-01-07

**Authors:** Shih-Wei Yang, Yun-Shien Lee, Liang-Che Chang, Cheng-Han Yang, Cheng-Ming Luo, Pei-Wen Wu

**Affiliations:** 1 Department of Otolaryngology-Head and Neck Surgery, Chang Gung Memorial Hospital, Keelung, No. 222, Mai Chin Road, Keelung, 204 Taiwan, Republic of China; 2 College of Medicine, Chang Gung University, Taoyuan, Taiwan, Republic of China; 3 Genomic Medicine Research Core Laboratory, Chang Gung Memorial Hospital, Taoyuan, Taiwan, Republic of China; 4 Department of Biotechnology, Ming Chuan University, Taoyuan, Taiwan, Republic of China; 5 Department of Pathology, Chang Gung Memorial Hospital, Keelung, Taiwan, Republic of China

**Keywords:** Leukoplakia, Tongue, Malignant transformation, Recurrence, Carbon dioxide laser

## Abstract

**Objectives:**

The tongue is identified as a high-risk site for oral leukoplakia and malignant transformation. The purpose of this study is to investigate the clinicopathological characteristics and treatment outcomes of tongue leukoplakia and assess the factors related to recurrence and malignant transformation.

**Materials and methods:**

One hundred and forty-four patients who received carbon dioxide laser surgery for tongue leukoplakia from 2002 to 2019 were analyzed statistically.

**Results:**

The follow-up period was 54.90 ± 54.41 months. Thirty patients showed postoperative recurrence (20.83%), and 12 patients developed malignant transformation (8.33%). The annual transformation rate was 2.28%. Univariate analysis showed that a history of head and neck cancer, size of lesion area, clinical appearance, and pathology were significant factors for both recurrence and malignant transformation. In the multivariate logistic regression, a history of head and neck cancer and size of lesion area were independent prognostic factors for recurrence, and a history of head and neck cancer was the only independent factor for postoperative malignant change.

**Conclusions:**

Clinicians should adopt more aggressive strategies for tongue leukoplakia patients with a history of head and neck cancer.

**Clinical relevance:**

These results may help clinicians gain a better understanding of oral tongue leukoplakia.

## Introduction

Oral squamous cell carcinoma (OSCC) accounts for about 3–5% of all the malignant tumors in the human body and affects more than 300,000 people worldwide annually; the incidence is higher in South Asian and Southeast Asian populations [1, 2], including regions endemic for the use of betel quid products [3, 4]. Radical surgeries with adequate peripheral and deep margins for oral cavity tumors and neck dissection are usually indicated for patients with OSCC. The oral cavity, which serves as a point of entry, a site for breakdown and tasting of food, and a sound resonance chamber, is unavoidably destroyed by surgery, possibly compromising the functions of swallowing, pronunciation, and chewing. Early diagnosis of OSCC is of paramount importance since patients who receive early treatment may show less functional morbidity, less cosmetic disfiguration, and lower cost of care and have a better outcome and higher quality of life postoperatively [5–7]. Although OSCC can develop de novo, various studies have established that most oral cancers are preceded by some visible clinical changes on the oral mucosa, or potentially malignant lesions [1, 3]. At a World Health Organization workshop in 2005, the term oral potentially malignant disorders (OPMDs) was suggested as a replacement for premalignant oral lesions and conditions [8]. In comparison with the normal oral mucosa, OPMDs, which encompass a number of lesion types, including leukoplakia, erythroplakia, oral lichen planus, oral submucous fibrosis, and other miscellaneous lesions, are characteristics of an increased risk of malignant transformation [1, 9, 10]. Oral leukoplakia (OLK) is the most common type of OPMD and has been widely studied [2, 5, 9–13]. The risks of malignant transformation of OLK have always been the focus of clinical attention. The risk factors for malignant transformation include female gender, old age, large size, location on the tongue or floor of mouth, non-homogeneous leukoplakia, a history of smoking/betel nut chewing, and high-grade dysplasia on initial biopsy [5, 10, 13–18]. Most studies on OLK were conducted in all parts of oral cavity mucosa, including the buccal area, tongue, gums, labial region, floor of mouth, palate, and retromolar mucosa. To the best of our knowledge, few studies on tongue leukoplakia have been conducted to date [13, 19, 20]. The aim of this study was to investigate the clinicopathological characteristics and treatment outcomes of oral tongue leukoplakia and analyze the factors related to the recurrence and malignant transformation of tongue leukoplakia treated by surgical excision with carbon dioxide (CO_2_) laser, including postoperative recurrence and malignant transformation.

## Materials and methods

This study was approved by the Institutional Review Board of Chang Gung Memorial Hospital (License No.: 201901384B0). Medical records of patients with OLK that received transoral laser excision at the Department of Otolaryngology of Keelung Chang Gung Memorial Hospital, from Sept 2002 to Oct 2019, were retrospectively reviewed. Information regarding patient enrollment, treatment, and the inclusion and exclusion criteria are listed in Table 1. Clinicopathological characteristics and factors related to the treatment outcomes, including gender, age, body mass index, history of radiotherapy, alcohol drinking, cigarette smoking, betel quid chewing, diabetes mellitus, metformin treatment, past history of head and neck cancer, clinical presentation, area of the lesion(s), pathological results, occurrence of leukoplakia at sites other than the tongue, *Candida* infection, and subsites of tongue, were analyzed. The history of betel quid chewing, alcohol consumption, and tobacco use were obtained by detailed questioning at the patients’ first visit to the outpatient department. The criteria for a positive assignment were at least 1 quid daily for at least 1 year for chewers of betel quid, at least 1 cigarette per day for at least 1 year for cigarette smokers, and drinking more than 4 days a week for at least 1 year for alcohol drinkers [21]. The CO_2_ laser used was an UltraPulse Encore (Lumenis®, Inc., Yokneam, Israel). A power setting of 8–10 W in a continuous-wave mode was chosen. Excision was conducted using a hand-held delivery device, and the spot size was adjusted to 1 mm in diameter. The hand piece was provided with a helium–neon aiming beam to facilitate targeting of the leukoplakia lesion. The outlines of the resection margins were situated at least 2–3 mm outside the targeted lesion to achieve adequate excision and obtain adequate tissue not affected by laser cauterization for histopathological diagnosis. The area of the leukoplakia was measured directly on the excised specimen.

**Table 1:** The protocol of patient enrollment, treatment, and the inclusion and exclusion criteria of patients

1. Enrollment of patients	
Thorough oral cavity examination by an otolaryngologist	
Written consent signed by every patient before surgery	
Preoperatively the types of leukoplakia [8, 10] photographed and later reviewed by two specialist of otolaryngology	
2. Carbon dioxide laser surgical intervention [21–23]	
Power setting 8–10 W, continuous-wave mode	
Laser spot size: 1 mm	
Excision with a surgical margin of 3 mm	
The excised wound left for secondary intention	
3. Pathological diagnosis	
All surgical specimens examined by 2 different pathologists	
Binary grading system of pathology by the WHO [24]	
4. Inclusion criteria	
Clinical diagnosis of leukoplakia on the mobile tongue and treated with CO_2_ laser	
All the lesions of leukoplakia were synchronous	
Patients’ age 20 or older	
5. Exclusion criteria	
Patients’ age younger than 20 years	
Other kinds of OPMDs, such as submucous fibrosis, lichen planus, and erythroplakia	
Previous treatment of tongue leukoplakia at other medical facilities	
Surgical margins involved by hyperkeratosis or dysplasia	
Pathology not available or no agreeable pathological diagnosis made	
Overt carcinoma on inspection	
Exophytic, papillary, warty, and verrucous appearance of proliferative verrucous leukoplakia	
Initial pathological diagnoses being carcinoma or malignancies	
Obvious ulceration	
Papilloma with a gross papillary appearance	
Treated by laser vaporization	

*OPMDs* oral potentially malignant disorders

Postoperative recurrence is defined as an OLK lesion that showed postoperative regrowth at the same site after confirmation of healing of the surgical wound(s) [25]. If tongue leukoplakia lesion appeared on a different site from the previous surgically treated location, it was defined as a new lesion instead of recurrence and this kind of case(s) was excluded. If a patient had one or more than one lesion of leukoplakia only on the mobile tongue, the case was defined as “single.” A “multifocal condition” described leukoplakia involving other parts of the oral mucosa in addition to the mobile tongue. In cases with more than one site of leukoplakia lesion, all the lesions were synchronous. The area of tongue leukoplakia in a patient was a summation of all tongue leukoplakia lesions if more than one lesion occurred. When the patient had more than one lesion, the highest degree of pathology and most severe form of morphology of oral tongue leukoplakia were documented for analysis and statistical calculation on a per capita basis.

All surgical procedures were conducted by one doctor (S.-W.Y.) under local anesthesia [21–23]. The postoperative follow-up was uneventful.

All the clinicopathological factors related to postoperative recurrence and malignant transformation were statistically analyzed in univariate analysis. The factors significantly related to postoperative recurrence and malignant transformation in the univariate analysis were further analyzed by the multivariate logistic regression model.

### Statistical analysis

Results were presented descriptively, with factors related to postoperative recurrence and malignant transformation of tongue leukoplakia. For univariate analysis, Fisher’s exact test and logistic regression were performed for discrete and continuous variables, respectively. For multivariate analysis, multiple logistic regression was performed. Odds ratio (OR) and 95% confidence intervals (CIs) were calculated using a two-tailed test of significance (*P* < 0.05) for each factor. Survival analyses were performed using Kaplan–Meier curves with log rank tests (for factor with two groups of subjects).

We made the following considerations: (1) if the 95% CI excludes the null value (1.0), and the *p* value of OR (or HR) of the risk factor must be < 0.05; (2) if the value of the OR (or HR) was greater than 1.0, the risk was increased; and (3) if the value was less than 1.0, the risk was reduced or indicated a protective effect. Fisher’s exact tests were calculated using the MATLAB version R2015a (Mathworks Inc., Natick, MA, USA). Kaplan–Meier curves with log rank tests and a multivariate logistic regression model using the Statistical Package SPSS version 22 (SPSS Inc., Chicago, IL, USA) were used to determine the distinct factors affecting postoperative recurrence and malignant transformation of oral tongue leukoplakia treated with CO_2_ laser.

## Results

### Clinicopathological characteristics

**Fig. 1:**
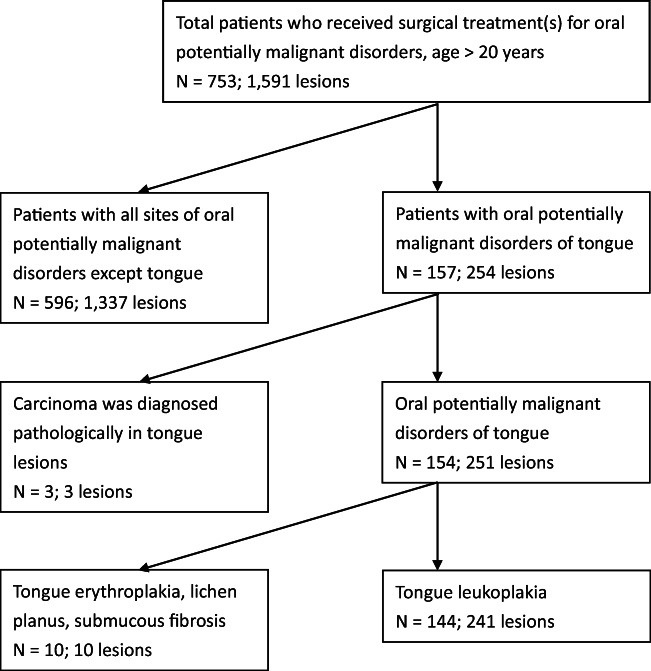
The algorithm for identifying study cohorts

Overall, 753 patients with 1591 OPMD lesions underwent CO_2_ laser surgery at the department from 2002 to 2019. Excluding patients with OPMDs not occurring on the oral tongue, initial diagnosis of carcinoma, and clinical tongue OPMDs other than leukoplakia, 144 patients with 241 lesions of tongue leukoplakia were enrolled (Fig. 1). Among the 144, 108 were male (75.0%) and 36 were female (25.0%), and their ages ranged from 25 to 83 years with a median age of 52.0 years and an average age of 52.17 ± 11.68 years. The average follow-up period was 54.9 ± 54.41 months. Multiple lesions occurred on the tongue or other sites of the oral cavity in some patients. Homogeneous and non-homogeneous leukoplakia could occur on different sites of the oral cavity or on the same site in the cases with postoperative recurrence. Pathological results of different severity also possibly occurred in the different or recurrent sites of lesions in a single patient. It is not possible to correlate every patient with a single morphological description or pathological findings unless the patient had only one leukoplakia lesion. Therefore, the more severe form of morphology and the highest degree of pathological severity of tongue leukoplakia were recorded on a per capita basis. In this study, 78 (54.17%) out of 144 patients had OLK at other sites in addition to tongue leukoplakia, or multifocal lesions such as buccal leukoplakia in 71 patients, retromolar leukoplakia in 16, gum leukoplakia in 9, labial leukoplakia in 5, floor of mouth leukoplakia in 3, and palate leukoplakia in 2. Among the 66 patients (45.83%) with only tongue leukoplakia, 53 patients had only 1 lesion of tongue leukoplakia during the cohort follow-up. Ninety-seven patients (67.36%) had homogeneous tongue leukoplakia and 47 (32.64%) had non-homogeneous tongue leukoplakia. The numbers of cases of pathologically squamous hyperplasia, mild dysplasia, moderate dysplasia, and severe dysplasia/carcinoma in situ (CIS) were 37, 62, 22, and 23, respectively. If a binary classification was adopted [24], high-risk lesions (45 cases, including moderate dysplasia and severe dysplasia/CIS) was outnumbered by low-risk lesions (99 cases, including squamous hyperplasia and mild dysplasia). The average area of tongue leukoplakia was 1.66 ± 1.84 cm^2^, median 1.11 cm^2^. There were 30 patients (20.83%) who showed postoperative recurrence and 12 patients (8.33%) showed postoperative malignant transformation of tongue leukoplakia. Among the 12 cases who developed malignant changes, 1 case had ventrolateral tongue cancer and buccal cancer, the other 11 cases had only tongue cancer, including 3 occurring on the dorsal tongue and 8 on the ventrolateral tongue. The time to develop recurrence and carcinoma was 3.62 ± 3.65 and 3.65 ± 2.54 years, respectively. The annual recurrence rate was 5.76%. The cumulative malignant transformation rate was 8.33% and annual transformation rate (ATR) was 2.28%. The demographic and clinicopathological data are shown in Table 2.

**Table 2:** Clinicopathological characteristics of patients who received laser surgery for tongue leukoplakia (*n* = 144)

	Case No.	Percent
Gender		
Female	36	25.00%
Male	108	75.00%
Age (mean ± standard deviation: 52.17 ± 11.72 years old)		
< 65	121	84.03%
≥ 65	23	15.97%
History of head and neck cancer		
No	111	77.08%
Yes	33	22.92%
History of radiotherapy		
No	131	90.97%
Yes	13	9.03%
Alcohol drinking		
No	93	64.58%
Ex-drinker	37	25.69%
Current drinker	14	9.72%
Smoking		
No	40	27.78%
Ex-smoker	44	30.56%
Current smoker	60	41.67%
Betel quid chewing		
No	74	51.39%
Ex-chewer	62	43.06%
Current chewer	8	5.56%
Diabetes mellitus^a^		
No	112	77.78%
Yes	30	20.83%
Metformin taken^b^		0.00%
No	114	79.17%
Yes	26	18.06%
Occurrence of leukoplakia in addition to tongue^c^		
No (single)	66	45.83%
Yes (multifocal)	78	54.17%
*Candida* infection^d^		
No	128	88.89%
Yes	16	11.11%
Subsites of tongue leukoplakia^d^		
Dorsal tongue mucosa	40	25.48%
Ventrolateral tongue mucosa	117	74.52%
Morphological outlooks		
Homogeneous	97	67.36%
Non-homogeneous	47	32.64%
Histopathological diagnosis		
Squamous hyperplasia	37	25.69%
Mild dysplasia	62	43.06%
Moderate dysplasia	22	15.28%
Severe dysplasia/carcinoma in situ	23	15.97%
Postoperative recurrence		
No	114	79.17%
Yes	30	20.83%
Postoperative malignant transformation		
No	132	91.67%
Yes	12	8.33%
Body mass index	27.26 ± 15.06	
Area (cm^2^) of the lesion(s)^e^	1.66 ± 1.84	
Cumulative malignant transformation rate	8.33%	
Duration of follow-up (months)	54.90 ± 54.41	
Annual recurrence rate^f^	5.76%	
Annual transformation rate^f^	2.28%	

^a^Two pieces of missing data in the group of diabetes mellitus (*n* = 142)

^b^Four pieces of missing data in the group of metformin taken (*n* = 140)

^c^If a patient has other sites of oral leukoplakia in addition to tongue, the patient will be categorized as “yes”

^d^Thirteen patients had both dorsal and ventrolateral tongue leukoplakia

^e^If the patient has more than 1 site of tongue leukoplakia, the area is the sum of all tongue leukoplakia lesions

^f^The annual recurrence rate and annual transformation rate is calculated by the recurrence rate and malignant transformation rate divided by the average time of development of recurrence or carcinoma (year)

### Univariate and multivariate analyses

**Fig. 2:**
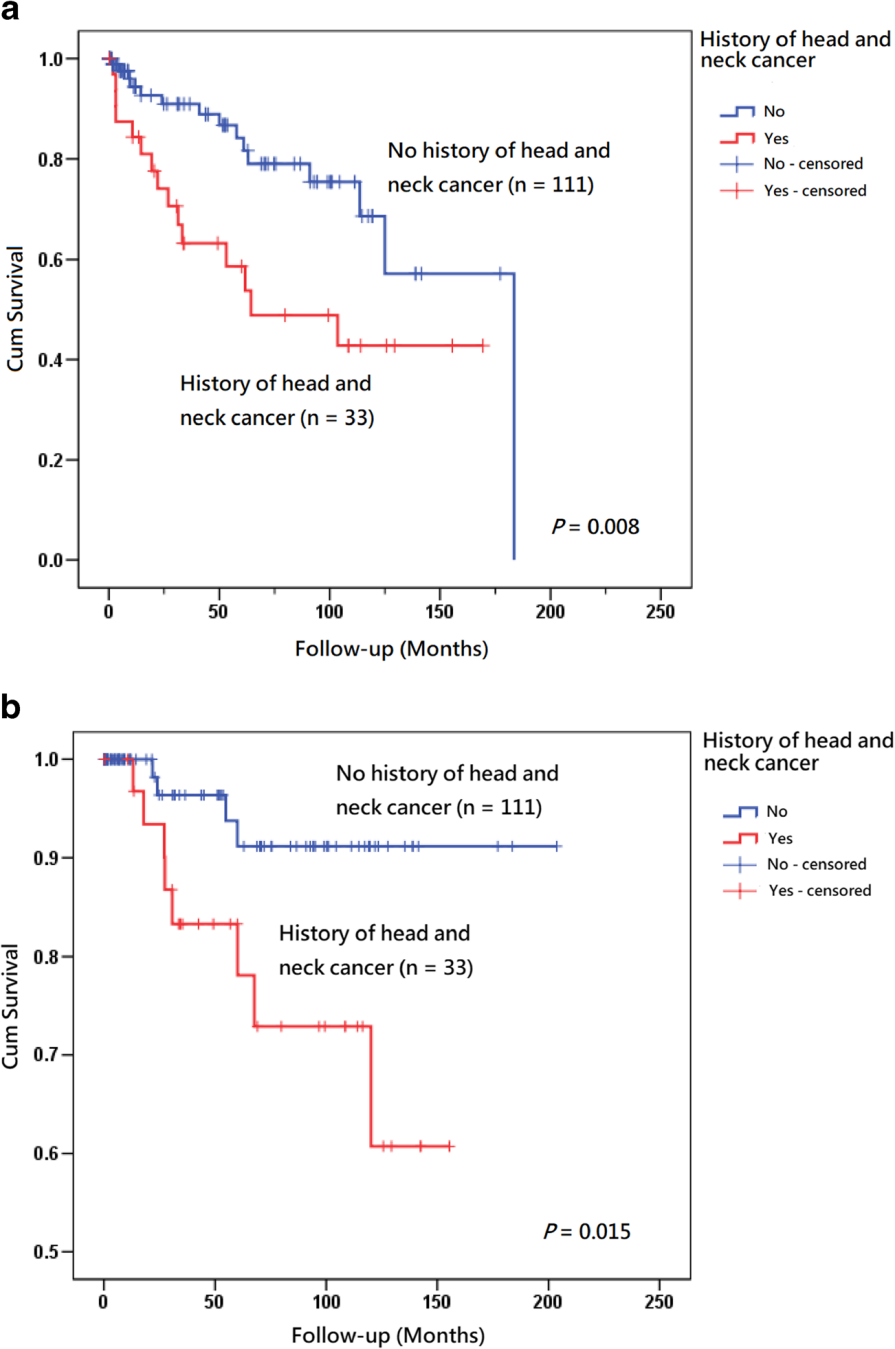
**a** Kaplan–Meier analysis with a log rank test of recurrence rate of tongue leukoplakia after carbon dioxide laser surgery according to patients without a history of head and neck cancer (*n* = 111) (blue line) versus without a history of head and neck cancer (*n* = 33) (red line). **b** Kaplan–Meier analysis with a log rank test of postoperative malignant transformation rate of tongue leukoplakia after carbon dioxide laser surgery according to patients without a history of head and neck cancer (*n* = 111) (blue line) versus with a history of head and neck cancer (*n* = 33) (red line)

**Fig. 3:**
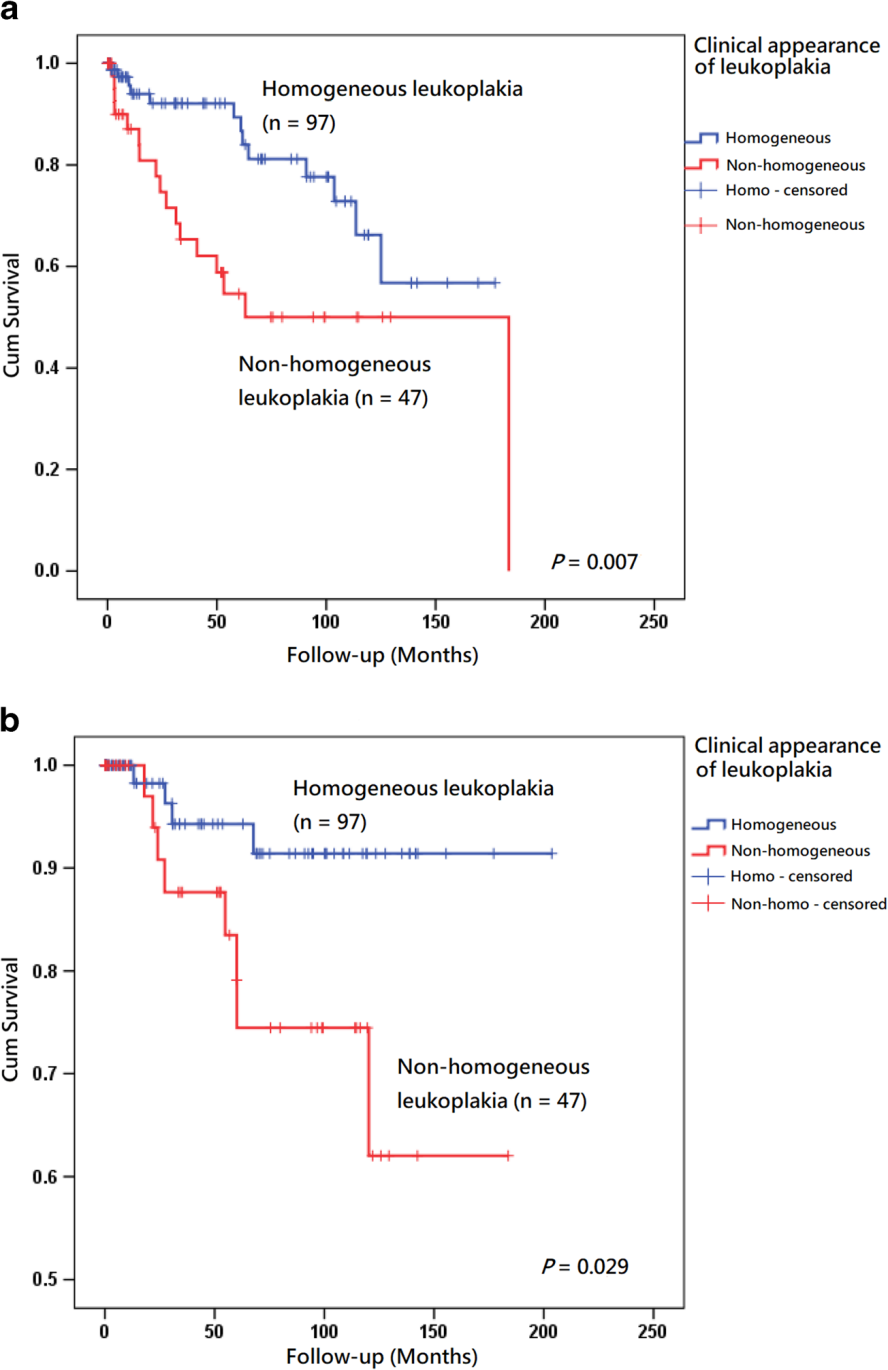
**a** Kaplan–Meier analysis with a log rank test of recurrence of tongue leukoplakia after carbon dioxide laser surgery in a subgroup of patients who had a homogeneous clinical appearance (*n* = 97) (blue line) versus those who had a non-homogeneous clinical appearance (*n* = 47) (red line). **b** Kaplan–Meier analysis with a log rank test of malignant change of tongue leukoplakia after carbon dioxide laser surgery in a subgroup of patients who had a homogeneous clinical appearance (*n* = 97) (blue line) versus those who had a non-homogeneous clinical appearance (*n* = 47) (red line)

**Fig. 4:**
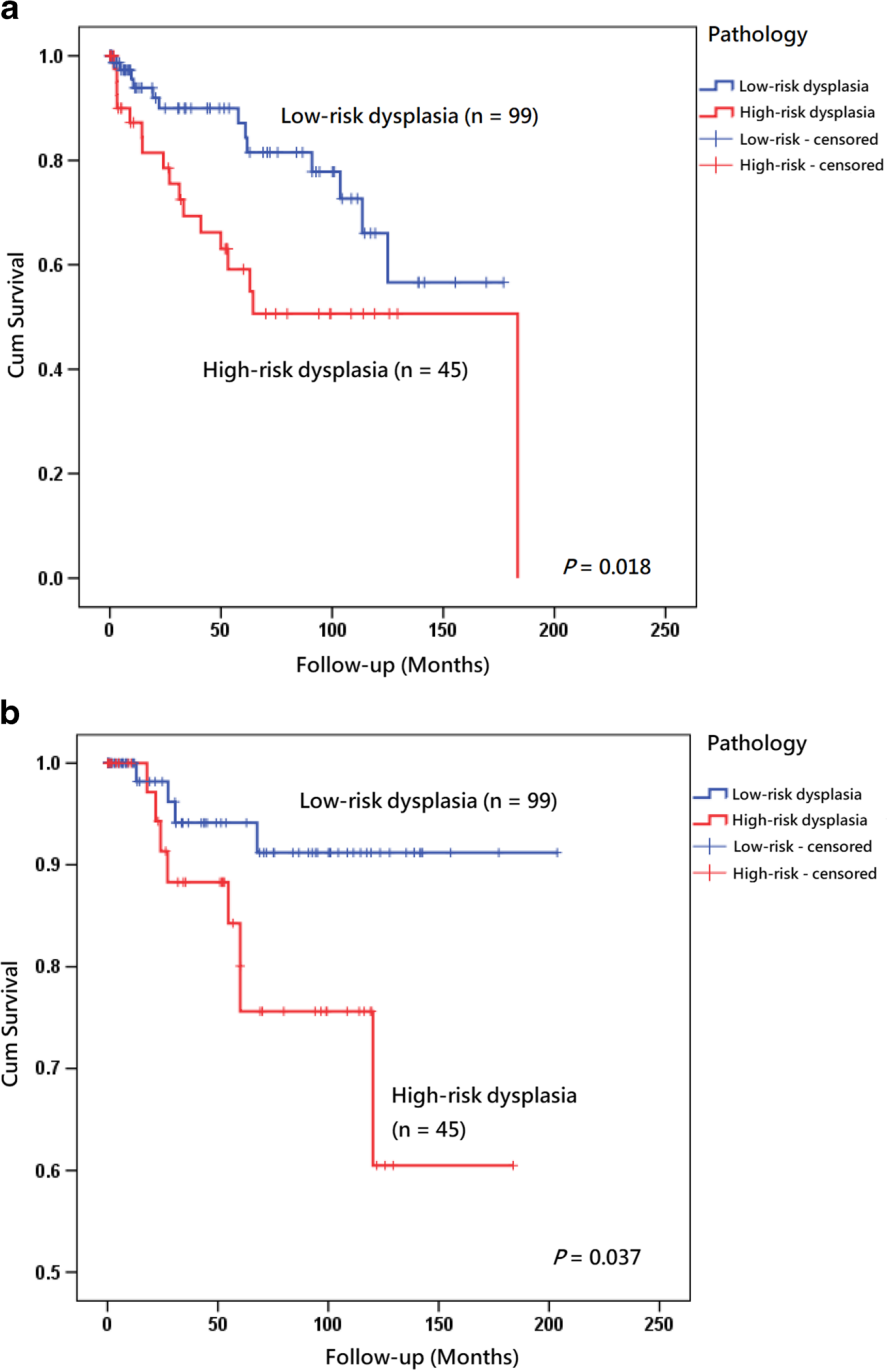
**a** Kaplan–Meier analysis with a log rank test of recurrence of tongue leukoplakia after carbon dioxide laser surgery between low-risk dysplasia (*n* = 99) (blue line) and high-risk dysplasia (*n* = 45) (red line). **b** Kaplan–Meier analysis with a log rank test of malignant change of tongue leukoplakia after carbon dioxide laser surgery between low-risk dysplasia (*n* = 99) (blue line) and high-risk dysplasia (*n* = 45) (red line)

**Fig. 5:**
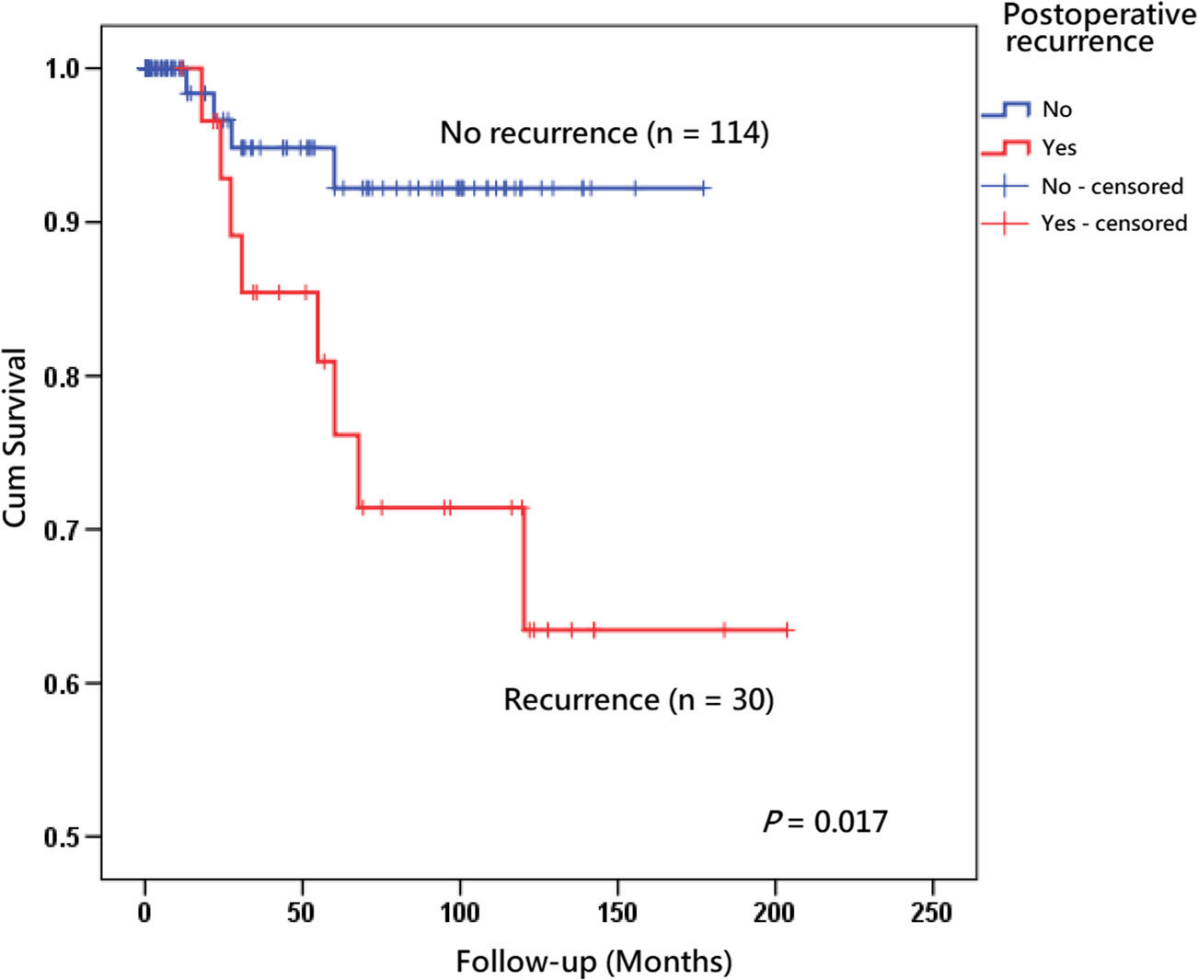
Kaplan–Meier analysis with a log rank test of malignant change of tongue leukoplakia after carbon dioxide laser surgery in a subgroup patients whose lesions did recur (*n* = 114) (blue line) versus whose lesions recurred (*n* = 30) (red line)

Past history of head and neck cancer, clinical presentation, area of the lesion(s), and pathology were significant risk factors associated with both postoperative recurrence and malignant transformation (Table 3, Figs. 2, 3, and 4). Gender, age, body mass index, history of radiotherapy, alcohol drinking, cigarette smoking, betel quid chewing, diabetes mellitus, metformin treatment, occurrence of leukoplakia at sites other than tongue, *Candida* infection, and subsites of tongue did not show statistical significance. In addition, postoperative recurrence was a significant associated factor related to postoperative malignant transformation (*P* = 0.017, Table 3, Fig. 5). The results of the multivariate analysis demonstrated that a history of head and neck cancer and area of the lesions were the 2 independent prognostic factors associated with recurrence and a history of head and neck cancer was the only independent prognostic factor associated with postoperative malignant transformation (Table 4).

**Table 3:** Univariate analysis of postoperative recurrence and malignant transformation of patients with tongue leukoplakia (*n* = 144)

	Postoperative recurrence				Postoperative malignant transformation			
	No (*n* = 114)	Yes (*n* = 30)	Odds ratio (95%confidence interval)	*P* value	No (*n* = 132)	Yes (*n* = 12)	Odds ratio (95%confidence interval)	*P* value
Gender				0.98				0.28
Female	28	8	1.0		34	2	1.0	
Male	86	22	1.10 (0.49–2.46)		98	10	2.36 (0.71–7.85)	
Age				0.87				0.90
< 65	96	25	1.0		111	10	1.0	
≥ 65	18	5	1.04 (0.38–2.89)		21	2	1.29 (0.24–6.79)	
Body mass index^a^	25.83 ± 4.54^b^	26.62 ± 4.62	1.04 (0.95–1.13)	0.40	26.1 ± 4.62^c^	24.91 ± 3.68	0.94 (0.81–1.09)	0.38
History of head and neck cancer				*0.034*				*0.013*
No	96	15	*1.0*		107	4	*1.0*	
Yes	18	15	*2.56* (*1.16–5.66*)		25	8	*5.96* (*1.71–20.77*)	
History of radiotherapy				0.70				0.91
No	104	27	1.0		121	10	1.0	
Yes	10	3	0.70 (0.25–2.01)		11	2	1.67 (0.28–9.97)	
Alcohol drinking				0.32				0.65
No	76	17	1.0		85	8	1.0	
Ex-drinker	26	11	1.89 (0.78 ~ 4.56)		33	4	1.29 (0.36 ~ 4.57)	
Current drinker	12	2	0.75 (0.15 ~ 3.64)		14	0	0.00 (0.00 ~ 65,535.00)	
Smoking				0.50				0.64
No	34	6	1.0		38	2	1.0	
Ex-smoker	35	9	1.46 (0.47 ~ 4.54)		39	5	2.44 (0.45 ~ 13.33)	
Current smoker	45	15	1.89 (0.66 ~ 5.38)		55	5	1.73 (0.32 ~ 9.37)	
Betel quid chewing				0.31				0.89
No	62	12	1.0		68	6	1.0	
Ex-chewer	46	16	1.80 (0.78 ~ 4.16)		56	6	1.21 (0.37 ~ 3.97)	
Current chewer	6	2	1.72 (0.31 ~ 9.58)		8	0	0.00 (0.00 ~ 65,535.00)	
Diabetes mellitus				0.94				0.90
No	91^d^	21^e^	1.0		103^f^	9	1.0	
Yes	22^d^	8^e^	1.14 (0.49–2.64)		27^f^	3	0.87 (0.24–3.19)	
Metformin taken				0.73				0.68
No	93^g^	21‖	1.0		104^c^	10	1.0	
Yes	18^g^	8‖	1.28 (0.54–3.06)		24^c^	2	0.60 (0.16–2.25)	
Occurrence of leukoplakia in addition to tongue				0.92				0.67
No (single)	54	12	1.0		60	6	1.0	
Yes (multifocal)	60	18	0.89 (0.42–1.88)		72	6	0.64 (0.20–2.09)	
*Candida* infection^g^				0.26				0.70
No	105	23	1.0		118	10	1.0	
Yes	9	7	2.07 (0.74–5.79)		14	2	1.01 (0.21–4.71)	
Subsites of tongue leukoplakia				0.15				0.29
Dorsal tongue: absent	87	17	1.0		98	6	1.0	
Dorsal tongue: present	27	13	1.94 (0.88–4.26)		34	6	2.36 (0.69–8.03)	
Subsites of tongue leukoplakia				0.48				1.00
Ventrolateral tongue: absent	22	5	1.0		25	2	1.0	
Ventrolateral tongue: present	92	25	1.51 (0.63–3.60)		107	10	1.30 (0.32–5.25)	
Morphological outlooks				*0.0079*				**0.030**
Homogeneous	84	13	*1.0*		93	4	*1.0*	
Non-homogeneous	30	17	*3.07* (*1.42 V 6.64*)		39	8	*4.62* (*1.37–15.55*)	
Area (cm^2^) of the lesion(s)^a^	1.35 ± 1.55	2.83 ± 2.34	*1.46* (*1.17–1.81*)	*0.001*	1.53 ± 1.60	3.04 ± 3.32	*1.35* (*1.06–1.70*)	*0.013*
Pathology				*0.019*				*0.040*
Low-risk lesion (hyperplasia and mild dysplasia)	86	13	*1.0*		95	4	*1.0*	
High-risk lesion (moderate dysplasia and severe dysplasia)	28	17	*2.66* (*1.25–5.68*)		37	8	*4.25* (*1.28–14.19*)	
Postoperative recurrence								*0.017*
No	NA	NA			110	4	*1.0*	
Yes	NA	NA			22	8	*5.39* (*1.58–18.39*)	

*NA* data not available

When the factor(s) is(are) statistically significant (*p* < 0.05), the number(s) is (are) presented in italic

^a^Univariate analysis was calculated by logistic regression for continuous predictor variables

^b^Four pieces of missing data in this group (*n* = 110)

^c^Four pieces of missing data in this group (*n* = 128)

^d^One piece of missing data in this group (*n* = 113)

^e^One piece of missing data in this group (*n* = 29)

^f^Two pieces of missing data in this group (*n* = 130)

^g^Three pieces of missing data in this group (*n* = 111)

**Table 4:** Multivariate analysis of postoperative recurrence and malignant transformation of patients with tongue leukoplakia (*n* = 144)

	Postoperative recurrence		Postoperative malignant transformation	
	Odds ratio (95% confidence interval)	*P* value	Odds ratio (95% confidence interval)	*P* value
History of head and neck cancer	*4.35* (*1.64–11.56*)	*0.003*	*4.57* (1*.10–19.09*)	*0.037*
Morphological outlooks	1.70 (0.41–7.02)	0.46	1.43 (0.17–12.44)	0.74
Area (cm^2^) of the lesion(s)	*1.45* (*1.14–1.84*)	*0.002*	1.20 (0.89–1.61)	0.23
Pathology	1.52 (0.37–6.34)	0.56	1.53 (0.18–13.43)	0.7
Postoperative recurrence	NA	NA	3.99 (0.93–17.13)	0.063
Constant	NA	0.095	NA	0.001

*NA* not available

^When the factor(s) is(are) statistically significant (*p* < 0.05), the number(s) is (are) presented in italic^

## Discussion

In the present study of oral tongue leukoplakia treated by CO_2_ laser excision, a history of head and neck cancer, size of the lesion area, clinical appearance, and pathology were found to be significant factors related to recurrence and malignant transformation. Postoperative recurrence itself was also a significant factor associated with malignant transformation. OLK is the most common OPMD [2, 5, 9–13], but it is still an enigmatic condition with regard to a successful treatment outcome and prediction of malignant transformation. Among the risk factors associated with malignant transformation of OLK, tongue has been a site of particular concern [5, 14, 15, 26, 27], including the ventral or lateral tongue [10, 13, 16, 28, 29], but few studies focusing solely on tongue leukoplakia have been conducted. In a study of 35 patients with tongue leukoplakia treated with surgery in Japan, CO_2_ laser was found to be an effective tool for tongue leukoplakia. However, no follow-up duration was recorded and no factors related to the treatment outcomes were analyzed [20]. The present series is the first to analyze the factors associated with postoperative recurrence and postoperative malignant transformation of tongue leukoplakia at both the dorsal and ventrolateral sites. A history of head and neck cancer, morphology, area, and pathology were significantly associated with the postoperative recurrence and malignant change. Besides, postoperative recurrence was also a significant factor related to postoperative malignant change. In the multivariate logistic regression analyses, a history of head and neck cancer and area were independent prognostic factors for postoperative recurrence and a history of head and neck cancer was the only independent prognostic factor for postoperative malignant transformation (Table 4).

The relationship between a history of head and neck cancer in the context of postoperative recurrence and malignant transformation has been studied in several studies [23, 29–32]. In a cross-sectional study of prevalence and risk factors of carcinoma and dysplasia in 1046 patients with OLK in Taiwan, a history of head and neck cancer was not a factor related to the presence of dysplasia or carcinoma in the pathological diagnosis of OLK [16]. In two other cohort studies of patients with OLK treated with CO_2_ laser, a history of head and neck cancer was not a factor associated with recurrence either [23, 32]. As for malignant transformation, in a study of 70 patients with OLK treated with a high-dose isotretinoin induction regimen (1.5 mg/kg/day) for 3 months and a 9-month maintenance therapy with either low-dose isotretinoin (0.5 mg/kg/day) or β-carotene (30 mg/day), a history of oral cancer was one of the significant predictive factors of cancer risks [33].

Exogenous factors, such as tobacco use, alcohol drinking, or betel quid chewing, or inherent factors, such as genetic aberrations, are all possible etiologies for OLK [34]. The risk factors associated with the occurrence of OLK are similar to those for oral cancers, including tobacco consumption, alcohol use, and betel quid chewing [15, 28]. In a study of 43 patients with OLK treated by CO_2_ laser in Australia, alcohol consumption was found to be a significant factor associated with recurrence [35]. In our previous study on 114 patients with OLK treated by CO_2_ laser, tobacco use and betel nut chewing were factors related to postoperative recurrence [23]. However, in other studies, alcohol drinking and cigarette smoking were not factors associated with recurrence of OLK [36–38]. As for the role of oral habits in the development of malignant changes of OLK, similar conflict between the published reports was also found; oral habits were significant factors in some academic works [5, 29, 39] and non-significant in others [21, 38, 40–42]. In the present study, oral habits were not factors related to the recurrence or malignant transformation of oral tongue leukoplakia. Regarding the etiopathogenesis of oral cancers related to cigarette smoking, alcohol drinking, and betel quid chewing [43], discontinuation of oral habits is still highly recommended for patients with tongue leukoplakia. Genetically altered epithelial cells might not be revealed by routine histopathological examinations, even in locations with normal histology [44, 45]. In addition, genetic alteration, which paves the path to neoplastic transformation, may precede marked phenotypic changes [4]. Studies investigating the molecular changes of multiple lesions with different pathological severities from benign hyperplasia to carcinoma in the same patient and multiple primary malignant tumors in the same patient have been performed previously. The results showed that the genetic alterations of OPMD and OSCC in the same patient were clonally related [46, 47]. From a study of 87 lesions in 83 patients, including benign squamous hyperplasia, dysplasia, carcinoma in situ, and carcinoma, the surrounding mucosa of the precancerous or carcinomatous lesions shared common genetic alterations and was found to arise from a single progenitor clone, indicating that the genetic molecular changes might be more extensive than what could be detected clinically or microscopically [48]. These molecular biological researches support the field cancerization concept and could account for the role of a history of head and neck cancer in the postoperative recurrence and malignant transformation in this study. In the present study, a history of head and neck cancer was a significant factor and independent prognostic factor associated with postoperative recurrence and malignant transformation (Table 2, Fig. 2a, b). Further investigations are warranted to shed more light on this issue.

Our previous work on the elderly patients (> 65 years) with OLK found that lesion area was one of the independent factors for postoperative recurrence. The cut-off area was 2.95 cm^2^ [49]. In a retrospective cohort study of 144 patients with OLK in Amsterdam, treatment with CO_2_ laser vaporization was done in 95 patients and observation in 49. Sixteen patients (11.1%) showed malignant transformation of OLK and a large size of the lesion (> 4 cm) was the only statistically significant predictor of malignant transformation (*P* = 0.034) [42]. In another research on 236 patients with 269 oral premalignant lesions [17], the authors proposed that a size exceeding 200 mm^2^ had a 5.4-fold increased risk of malignant development after surgery. In addition, widespread leukoplakia, or multiple leukoplakia in multiple sites, and large confluent potentially malignant lesions that extended over more than one anatomical site were found to be significant factors in determining the risk of transformation to OSCC [4, 50]. In this series, the size or area of tongue leukoplakia was a significant factor related to postoperative recurrence (2.83 ± 2.34 cm^2^ vs. 1.35 ± 1.55 cm^2^) and malignant transformation (3.04 ± 3.32 cm^2^ vs. 1.53 ± 1.60 cm^2^) and also an independent prognostic factor for recurrence (Table 3). Patients with larger OLK were also reported to have poorer scores in the quality of life questionnaire evaluation [51]. Thus, physicians should pay more attention and adopt a more delicate and robust strategy in treating large area of tongue leukoplakia and postoperative follow-ups.

The outlooks of OLK can be divided into 2 major categories: homogeneous leukoplakia and non-homogeneous leukoplakia. The description and criteria are the same as mentioned in previous studies [9, 21]. Non-homogeneous OLK was a predictive factor that tended to be associated with recurrence in the Kaplan–Meier survival analysis model in our previous work (*P* = 0.029) [23]. Similar findings indicating that non-homogeneous OLK was more often related to postoperative recurrence were also found [36, 52]. Non-homogeneous leukoplakia has been proposed to be a strongly associated factor with an increased risk of malignant development of OPMDs, including OLK [7, 10, 18, 28, 47, 53–55]. In our earlier study of 114 patients with OLK treated with CO_2_ laser, non-homogeneous OLK was a significant predictor for malignant transformation in the Kaplan–Meier survival analysis model [21]. According to another study of 31 patients with homogeneous OLK, 34 with non-homogeneous OLK and 12 with erythroplakia treated with CO_2_ laser in London, non-homogeneous OLK was more often associated with post-treatment malignant transformation [52]. However, a contradictory result was obtained in a meta-analysis of malignant change of OLK treated with CO_2_ laser. The results demonstrated that the rate of malignant transformation was 5.78% in homogeneous OLK and 5.35% in non-homogeneous OLK. The authors advocated that evidence was still lacking in terms of a relationship between malignant transformation and risk factors of OLK patients managed with CO_2_ laser [41]. In this series, non-homogeneous tongue leukoplakia was a significant predictive factor for more recurrence and higher malignant transformation rate after surgical excision than the homogeneous type (Table 3, Fig. 3a, b). Non-homogeneous OLK was at a higher risk of harboring dysplasia and carcinoma [16]. After reviewing host and biologic factors, the ratio of patients showing a history of head and neck cancer (19/47 vs. 14/97, *P* = 0.0007, data not shown), the area of tongue leukoplakia (2.10 ± 2.23 vs. 1.45 ± 1.58 cm^2^, *P* = 0.043, data not shown), and the ratio of patients showing high-risk dysplasia (39/47 vs. 6/97, *P* < 0.0001, data not shown) in the tongue non-homogeneous leukoplakia in this study were higher than those in the homogeneous lesions, which might reflect the disease severity and explain why the treatment outcome of non-homogeneous leukoplakia was poorer than that of homogeneous leukoplakia. Non-homogeneous appearance is an ominous sign for tongue leukoplakia and should be managed aggressively.

Pathological examination is the standard diagnostic process and is essential for all cases of OLK because the lesions may contain foci of OSCC [13, 16, 44, 56], which indicates prompt definite treatment. Pathological demonstration of dysplasia is another important issue for OLK. The occurrence of dysplasia in an OLK lesion indicates a high probability of postoperative recurrence [23, 49] or malignant transformation [21, 54, 55, 57] in several studies. However, there were results contradictory to the concept, and those authors thought that the presence of any degree of epithelial dysplasia did not have any influence on the risk of postoperative recurrence [35, 36, 38] or malignant development [17, 44]. According to a study of 368 patients with oral epithelial dysplasia from Australia, 4.1% of cases with mild dysplasia showed malignant development and the severity of epithelial dysplasia was not associated with the risk of malignant transformation; therefore, complete excision of all the epithelial lesions with different degrees of dysplasia was suggested [58]. Since there is still no consensus in the literature concerning the relationship between malignant transformation and risk factors and considering the lack of any proven biomarkers in large cohort studies, histopathological grading of dysplasia is regarded as the gold standard in treating these patients [10]. In the present study, high-risk dysplasia, including moderate and severe dysplasia and carcinoma in situ, was a significant predictive factors for recurrence and malignant transformation (*P* < 0.05; Table 3, Fig. 4a, b). We think clinicians should remain alert to the presence of dysplasia, especially high-risk dysplasia, considering its relationship to postoperative recurrence and malignant transformation.

Thirty patients out of 144 showed recurrence after surgical excision in the present study. The recurrence rate was 20.83% and the annual recurrence rate was 5.76%. The status of postoperative recurrence of tongue leukoplakia was consistent with that of other studies of OLK whose annual recurrence rates were approximately 5–10% [23, 38, 59]. Postoperative recurrence was a significant associated factor and independent prognostic factor for malignant transformation in our previous study [21]. Besides, recurrence was also significantly associated with cancer transformation (*P* < 0.001) in a prospective longitudinal multicenter study of 180 patients who underwent surgical removal of OLK [36]. In the present study, recurrence itself was found to be a significant predictive factor for postoperative malignant transformation (*P* = 0.017; Table 2, Fig. 5), but not an independent prognostic predictor in the multivariate logistic regression model.

The cumulative malignant transformation rate and ATR of the mobile tongue leukoplakia were 8.33% and 2.28%, respectively (Table 2). In a meta-analysis of malignant transformation of OLK, 24 studies were audited and the overall rate of malignant transformation of OLK treated with CO_2_ laser was 4.5% [41]. In another systematic review of 24 articles on malignant transformation of oral leukoplakia, the estimated overall malignant transformation rate was 3.5% [60]. The timing of malignant development of OLK is unpredictable [26]. In this regard, the follow-up periods of the published studies might be different, and we think that the annual transformation rate is scientifically more reasonable than the overall cumulative transformation rate. ATR was not frequently investigated in the literature [42, 47, 61] and all the published studies were conducted on all sites of the oral cavity. The differences in treatment types among those studies, including surgical removal, medical treatment, or biopsies alone, might make the basis of comparison less robust. The follow-up time varied from 2.42 to 4.75 years. The ATR ranged from 1.2 to 2.6%. From the perspective of ATR, 2.28% for tongue leukoplakia in the present study did not seem to be higher than the other studies of OLK.

Proliferative verrucous leukoplakia (PVL), which has high rates of recurrence and malignant change, is a unique subtype of OLK. Clinically, the diagnosis of PVL is consistently a challenge. In addition to the criteria proposed by Hansen et al. in 1985 and Villa et al. in 2018 [62, 63], observation of the recurrent and aggressive clinical behaviors and the potential of malignant development of the lesion may be of help in diagnosing PVL. We tried to prevent PVL cases from entering the present study, so we recruited the cases of multifocal OLK patients whose lesions were synchronous and excluded the cases with new lesions of OLK after treatment and the cases with exophytic, papillary, warty, and verrucous appearance of OLK (Table 1).

There are some limitations in this study. First, some missing data were found in some of the variables due to their retrospective nature. Second, although we chose excision of the whole leukoplakia lesion instead of vaporization, the quality of histopathological diagnosis on the tissue might have been altered due to the thermal injury induced by the laser. If the pathologists could not make an agreeable pathological diagnosis, the case(s) would be excluded. Third, the sample size of oral tongue leukoplakia was relatively small. In our experience, it is difficult to enroll a large number of patients in a single-center facility. Large-scale, multicenter, and prospective cohort studies are warranted to further investigate the disease.

## Conclusions

For oral tongue leukoplakia, clinicians should adopt more aggressive treatment strategies for patients with a history of head and neck cancer.
